# Total fluid intake of children and adolescents: cross-sectional surveys in 13 countries worldwide

**DOI:** 10.1007/s00394-015-0946-6

**Published:** 2015-06-18

**Authors:** Iris Iglesia, Isabelle Guelinckx, Pilar M. De Miguel-Etayo, Esther M. González-Gil, Jordi Salas-Salvadó, Stavros A. Kavouras, Joan Gandy, Homero Martínez, Saptawati Bardosono, Morteza Abdollahi, Esmat Nasseri, Agnieszka Jarosz, Guansheng Ma, Esteban Carmuega, Isabelle Thiébaut, Luis A. Moreno

**Affiliations:** GENUD (Growth, Exercise, NUtrition and Development) Research Group, Faculty of Health Sciences, Universidad de Zaragoza, Zaragoza, Spain; Hydration & Health Department, Danone Research, Palaiseau, France; Human Nutrition Unit, Hospital Universitari de Sant Joan de Reus, Faculty of Medicine and Health Sciences, IISPV (Institut d’Investigació Sanitària Pere Virgili), Biochemistry Biotechnology Department, Universitat Rovira i Virgili, Reus, Spain; CIBERobn (Centro de Investigación Biomédica en Red Fisiopatología de la Obesidad y Nutrición), Institute of Health Carlos III, Madrid, Spain; Department of Health Human Performance and Recreation, University of Arkansas, Fayetteville, AR USA; British Dietetic Association, Birmingham, UK; School of Life and Medical Services, University of Hertfordshire, Hatfield, UK; RAND Corporation, Santa Monica, CA USA; Hospital Infantil de Mexico Federico Gomez, Mexico City, Mexico; Department of Nutrition, Faculty of Medicine, Universitas Indonesia, Jakarta, Indonesia; Department of Nutrition Research, National Nutrition and Food Technology Research Institute, Faculty of Nutrition Sciences and Food Technology, Shahid Beheshti University of Medical Sciences, Tehran, Iran; National Food and Nutrition Institute, Warsaw, Poland; National Institute for Nutrition and Food Safety, Chinese Center for Disease Control and Prevention, Beijing, China; Department of Nutrition and Food Hygiene, School of Public Health, Peking University, Beijing, China; Centro de Estudios Sobre Nutrición Infantil, Buenos Aires, Argentina; Research Centre of Epidemiology, Biostatistics and Clinical Research, School of Public Health, Université Libre de Bruxelles, Brussels, Belgium; Club Européen des Diététiciens de l’Enfance, Brussels, Belgium

**Keywords:** Fluid intake, Children, Adolescents, Worldwide, Dietary assessment, Hydration

## Abstract

**Purpose:**

To describe total fluid intake (TFI) according to socio-demographic characteristics in children and adolescents worldwide.

**Methods:**

Data of 3611 children (4–9 years) and 8109 adolescents (10–18 years) were retrieved from 13 cross-sectional surveys (47 % males). In three countries, school classes were randomly recruited with stratified cluster sampling design. In the other countries, participants were randomly recruited based on a quota method. TFI (drinking water and beverages of all kinds) was obtained with a fluid-specific record over 7 consecutive days. Adequacy was assessed by comparing TFI to 80 % of adequate intake (AI) for total water intake set by European Food Safety Authority. Data on height, weight and socio-economic level were collected in most countries.

**Results:**

The mean (SD) TFI ranged from [1.32 (0.68)] to [1.35 (0.71)] L/day. Non-adherence to AIs for fluids ranged from 10 % (Uruguay) to >90 % (Belgium). Females were more likely to meet the AIs for fluids than males (4–9 years: 28 %, OR 0.72, *p* = 0.002; 10–18 years: 20 %, OR 0.80, *p* = 0.001), while adolescents were less likely to meet the AI than children (OR 1.645, *p* < 0.001 in males and OR 1.625, *p* < 0.001 in females).

**Conclusions:**

A high proportion of children and adolescents are at risk of an inadequate fluid intake. This risk is especially high in males and adolescents when compared with females or children categories. This highlights water intake among young populations as an issue of global concern.

**Electronic supplementary material:**

The online version of this article (doi:10.1007/s00394-015-0946-6) contains supplementary material, which is available to authorized users.

## Introduction

Water is essential for life to the extent that hydration is a major key to survival [24]. This is especially true for infants and adolescents who have relatively high requirements for water to maintain an adequate body composition [28]. This high requirement can partially be explained because children have proportionally higher body water content than adults [5]. Besides the higher body water content, body surface area to body mass ratio is higher in children when compared with adults. This difference levels out by adolescence, when children have almost reached their adult size [27]. At this time, gender differences start to appear: females store more adipose tissue than males and therefore water percentages are lower than in males [1].

Dehydration (body water deficit) is a physiologic state that can have profound implications for human health [10]. Under conditions of severe dehydration, a decreased sympathetic nervous activity, impaired thermoregulation and impaired cognitive and physical performance can be observed [29]. In children, even mild dehydration can affect cognitive school performance [16, 17].

International organizations have set dietary reference intakes (DRIs) for total water in young populations, using different methodologies. For instance, in children between the ages of 4 and 13, the European Food Safety Authority (EFSA) based its DRIs primarily upon energy intake, while for adolescents aged 14 years through to adulthood, intake reference values are based on population median water consumption and desirable urine osmolality [1]. The Institute of Medicine (IOM) DRIs for total water intake in the USA and Canada are the median intakes observed in the National Health and Nutrition Examination Survey III (NHANES), for children aged 1–18 years as well as for adults [2]. Due to such differences in methods for the derivation of reference values, health authorities stress the necessity to establish the water intake recommendations based on water balance [1].

Despite the availability of intake reference values specific for children and adolescents, little is known about the adherence to these recommendations in these age groups. The limited data available suggest that children and adolescents do not drink enough and do not meet the daily recommended fluid intake. Total fluid intake (TFI) is set as the sum of liquids provided by all types of fluids or beverages, and it is assumed to be around 80 % of all the water intakes (20 % from foods) [1]. A German longitudinal study conducted in children and adolescents reported a mean total water intake of 1642 and 1457 ml in 9–13-year-old males and females, respectively [4]. This is approximately 450 ml lower than the corresponding EFSA reference values of 2100 (males) and 1900 ml (females) [26]. A similar situation was observed in European adolescents, as their mean total water intake was 1611 and 1316 ml in 12.5–17.5-year-old males and females, respectively [15]. In US children and adolescents, 4–19 years in the NHANES survey 2005–2006, mean daily total water intake was lower than the IOM adequate intake (AI) (only 15–35 % met the recommendations according to sex and age groups) [21]. In a Brazilian study [18], in which no statistically significant differences were observed between males and females, TFI was higher than that observed in the previously cited studies, being approximately 1750 ml for children and 2050 ml for adolescents.

The aim of the present study is to describe fluid intake and its variation by age (children 4–9 years vs. adolescents 10–18 years) and/or sex in 13 countries worldwide controlling for BMI and socio-economic status.

## Methods

### Design and study population

Cross-sectional surveys were identified from 13 different countries to summarize country-specific TFI of participants aged 4–18 years. The surveys (whose aim was to systematically obtain a complete set of data from fluid intake) were conducted during one period of the year in Latin America (Mexico, Brazil, Argentina, Uruguay), Europe (Spain, France, Belgium, UK, Poland, Turkey) and Asia (Iran, China, Indonesia) by private research organizations, the Université libre de Bruxelles/the Club Européen des Diététiciens de l’Enfance (CEDE), the Iranian National Nutrition & Food Technology Research Institute (NNFTRI) and the Chinese Center for Disease Control (CDC). The individual surveys took place between 2008 and 2014 (Online Resource 1).

The protocol of the published surveys was described in detail elsewhere [13, 14, 25], but will again briefly be described. The surveys performed in Belgium, Iran and China had a comparable method of recruitment: they recruited entire school classes via a random, stratified cluster sampling. In Belgium, 13 schools had accepted to participate, and in each school, the classes of the third- up to the sixth-grade participated [25]. In Iran, the recruitment was performed in Tehran. To cover all SES groups, Tehran was divided into three major areas: north, middle and south, representing high, middle and low SES, respectively. Eighteen schools were randomly selected to cover three school levels and two genders in every area. In selected schools, one class was randomly selected from each grade (except for the first and 12th grades). All of the students of a class were recruited. In China, a multi-stage random sampling method is adopted throughout the survey [13, 14]. The parents of the recruited school children were informed via meetings, written information sheets or phone calls. The surveys performed in the 10 other countries randomly recruited participants using a quota-based method with quotas set for age, gender, region of the country, habitat and/or socio-economic characteristics. The parents of the children were contacted via a database of individuals volunteering for population surveys or via a systematic door-to-door approach, with an invitation for their child to participate. Having a parent working in advertising, marketing, market research, media or manufacture, distribution and sale of beverages (in order to have participants that were not specifically aware of their fluid intake) and being incapable of completing the questionnaire in the language presented were exclusion criteria. Having a specific diagnosed disease and/or following a medically prescribed diet were additional exclusion criteria (to avoid individuals that might have modified their usual fluid intake) in the surveys of UK and China. The surveys in Argentina, Poland and Japan also excluded participants who took part in a survey about non-alcoholic drinks in the last 6 months.

All parents and children willing to participate (those who did not were not tracked) in the survey received detailed information about the survey objectives, what was expected from them, as well as a disclosure of the survey’s provisions to preserve confidentiality, risks and benefits, and a clear explanation about their option to voluntarily participate or not in the survey. After offering a full-informed description of the survey, parents were asked for their oral approval to let their child participate. No monetary incentive was offered for taking part in the survey. All data were recorded in an anonymous way. Therefore, participants cannot be identified, directly or through identifiers linked to the participants. The protocol of the unpublished surveys was reviewed and approved by the Institutional Review Board, Office of Research Compliance of the University of Arkansas (IRB Protocol # 14-12-376).

### Assessment of fluid intake

A fluid-specific record over 7 consecutive days was completed by the participants. These 7-day fluid-specific records and the associated written information were presented to the participants in the official language of the country and were in a paper format, except in France where the fluid records were completed online. This online fluid-specific record, which had the same structure as the paper version, was supported with paper memory cards to make notes throughout the day. An investigator delivered and explained the fluid record to the participants during a face-to-face interview at home. For children below 12 years, the primary caregiver of the child was asked to complete the fluid records. The researcher visited the home again after 7 days to collect the fluid-specific records and to ensure their completion. The surveys performed in Belgium, Iran and China deviated from this protocol as they recruited children in school classes [3, 5]. In these cases, both parents and teachers were involved in the completion of the fluid records.

The 7-day fluid-specific records in all surveys were structured in the same way in order to capture the type of fluid consumed, the volume of intake, the reasons, the moment of the day and the place of all fluid intakes. To assist the participants in estimating the consumed volumes, the records were supported by a photographic booklet of standard containers of fluids and in China by an additional scaled water container. The sum of all fluid types recorded was defined as TFI. To evaluate the adequacy of fluid intake, the EFSA age- and gender-specific reference values for total water intake were used in order not to overestimate the size of the problem of inadequate fluid intake (in comparison with other reference values), after extracting 20 % supposed to correspond to water intake from foods [1]. Consequently the reference values of 1.3, 1.7 and 2 L/day were used for males aged 4–8, 9–13 and 14–18 years, respectively. The reference values of 1.3, 1.5 and 1.6 L/day were used for females aged 4–8, 9–13 and 14–18 years respectively. These values will be referred to as EFSA AIs for fluids. However, the presentation of the results throughout the paper will be done only by children and adolescents, but fitting them in their corresponding reference value for water intake recommendations.

### Assessment of body composition and socio-economical level

Body mass index (BMI) serves as a proxy for both low physical activity and poor health status, two key determinants of water requirements. Height in metres (m) and weight in kilograms (kg) were self-reported in the surveys in Spain, France, UK and Turkey and measured by the investigator in the surveys in Belgium, Poland, Iran and China. No anthropometric data were collected in Mexico, Brazil, Uruguay, Argentina and Indonesia. Body mass index (BMI) was calculated (kg/m^2^) and categorized using the International Obesity Task Force cut-off points [8, 9]. These cut-off points were established according to thinness, sex and age (i.e. male, 8 years, BMI 14.14 kg/m^2^ or lower = underweight; male, 8 years, BMI = 14.15–18.43 kg/m^2^ = normal weight; male, 8 years, BMI = 18.44–21.59 kg/m^2^ = overweight; male, 8 years, BMI = 21.60 kg/m^2^ or higher = obesity). Socio-economic level (SEL) was assessed using a self-administered questionnaire in most of the countries and was categorized using the Market Research Society classification. This classification was based on the occupation of the chief income earner in the household [3, 12].

### Statistical analysis

Participants who did not complete the full 7-day fluid intake records or participants reporting a mean total daily fluid intake below 0.4 L/day or higher than 4 L/day were excluded from the analysis. The final sample size for this analysis was 11720 participants. Continuous and categorical data were, respectively, presented as mean (SD) and/or median (25th–75th percentile) and percentage (*n*). Standard error of the mean (SEM) and additional percentiles (5th, 10th, 90th, 95th) are reported in Online Resource 2. The effect of gender on the non-adherence to AIs for total water intake was tested with Chi-square test stratified by country.

Associations between compliance with AIs for fluids by gender and age group were assessed by logistic regression analysis (Fig. 2) stratifying the results by country. To estimate the strength of the association, odds ratios and 95 % confidence intervals (CI) were assessed using age- or gender-stratified forest plots. The models were adjusted for gender, BMI and SEL. Males were considered as reference in the first model (Fig. 2). Indonesia, Mexico, Argentina, Brazil and Uruguay were excluded from the logistic regression analysis due to the lack of data for some covariates. Analyses were performed using SPSS software version 20.0 (SPSS Inc, Chicago, IL). All statistical tests were two-tailed, and the significance level was set at *p* < 0.05.

## Results

### Sample description

The general characteristics of both male and female subjects are presented by country in Tables 1 and 2. A total of 5766 males (11.2 ± 3.3 years) and 6333 females (11.4 ± 3.3 years) were included in this study. In only 8 out of 13 countries, anthropometric data were obtained, and consequently the BMI of only 3934 males and 4498 females was calculated, and 64.5 % males and 67.8 % females of these were classified as normal weight. Medium socio-economic class was the most common SEL (44.0 % males and 47.0 % females).

**Table 1:** General characteristics of the male study population, categorized by country

	Sample size, *n* (% relative females)	Age, mean (SD)	Age categories, % (*n*)		BMI, mean (SD)	BMI classification^a^, % (*n*)				Socio-economic level^c^, % (*n*)		
			4–9 years	10–18 years		Underweight	Normal	Overweight	Obesity	AB	C	D
Mexico	406 (59)	9.0 (3.5)	58 (234)	42 (172)	n.a.	n.a.	n.a.	n.a.	n.a.	5 (21)	39 (157)	56 (228)
Brazil	395 (51)	10.3 (4.2)	46 (183)	54 (212)	n.a.	n.a.	n.a.	n.a.	n.a.	22 (87)	44 (172)	34 (136)
Uruguay	68 (49)	10.1 (3.7)	44 (30)	56 (38)	n.a.	n.a.	n.a.	n.a.	n.a.	31 (21)	28 (19)	41 (28)
Argentina	74 (38)	11.2 (4.5)	41 (30)	59 (44)	n.a.	n.a.	n.a.	n.a.	n.a.	27 (20)	32 (24)	41 (30)
Spain	106 (53)	10.5 (4.0)	41 (43)	59 (63)	19.4 (4.0)	9.4 (10)	59.4 (63)	25.5 (27)	5.7 (6)	25 (27)	42 (45)	32 (34)
France	211 (53)	8.9 (3.4)	56 (119)	44 (92)	17.6 (3.5)	18.0 (38)	63.8 (134)	15.2 (32)	2.9 (6)	n.a.	n.a.	n.a.
Belgium	375 (45)	10.3 (1.3)	31 (116)	69 (259)	18.4 (3.4)	6.3 (23)	69.6 (256)	17.7 (65)	6.5 (24)	n.a.	n.a.	n.a.
UK	157 (44)	10.0 (3.5)	43 (67)	57 (90)	21.2 (6.2)	8.6 (10)	41.7 (48)	27.0 (31)	22.6 (26)	16 (25)	46 (73)	38 (59)
Poland	170 (52)	10.1 (4.1)	47 (80)	53 (90)	18.1 (3.8)	3.7 (1)	77.8 (21)	14.8 (4)	3.7 (1)	8 (13)	65 (110)	28 (47)
Turkey	67 (18)	13.0 (4.1)	24 (16)	76 (51)	20.9 (3.9)	3.0 (2)	70.1 (47)	11.9 (8)	14.9 (10)	n.a.	n.a.	n.a.
Iran	367 (47)	12.8 (2.9)	23 (84)	77 (283)	20.8 (4.9)	9.3 (33)	56.9 (203)	21.6 (77)	12.3 (44)	32 (119)	29 (108)	38 (140)
China	2705 (48)	12.1 (2.6)	20 (540)	80 (2165)	19.1 (3.7)	11.1 (298)	65.8 (1766)	17.2 (461)	5.9 (159)	n.a.	n.a.	n.a.
Indonesia	443 (44)	10.2 (3.9)	45 (200)	55 (243)	n.a.	n.a.	n.a.	n.a.	n.a.	26 (117)	57 (252)	17 (74)
Total population ^b^	5766 (48)	11.2 (3.3)	32 (1844)	68 (3922)	19.2 (4.0)	10.5 (415)	64.5 (2538)	17.9 (705)	7.0 (276)	21 (450)	44 (960)	35 (776)

Data are presented as percentage (n) or mean (95 % CI)

*n.a.* not available, *BMI* body mass index

^a^BMI (kg/m^2^) classification according to IOTF guidelines [8, 9]

^b^Only countries with available data on the presented characteristics were included

^c^Category AB represents individuals with professional/managerial occupations, C represents individuals with other non-manual occupations and individuals having skilled manual occupations, and D represents individuals with semi-/unskilled manual occupations and people dependent on state benefits

**Table 2:** General characteristics of the female study population, categorized by country

	Sample size, *n* (% relative males)	Age, mean (SD)	Age categories, % (*n*)		BMI, mean (SD)	BMI classification^a^, % (*n*)				Socio-economic level^c^, % (*n*)		
			4–9 years	10–18 years		Underweight	Normal	Overweight	Obesity	AB	C	D
Mexico	287 (41)	9.4 (3.7)	53 (153)	47 (134)	n.a.	n.a.	n.a.	n.a.	n.a.	3 (8)	41 (119)	56 (160)
Brazil	384 (49)	10.3 (4.2)	43 (166)	57 (218)	n.a.	n.a.	n.a.	n.a.	n.a.	20 (75)	47 (181)	33 (128)
Uruguay	71 (51)	10.9 (4.0)	42 (30)	58 (41)	n.a.	n.a.	n.a.	n.a.	n.a.	27 (19)	25 (18)	48 (34)
Argentina	119 (62)	10.1 (4.2)	50 (59)	50 (60)	n.a.	n.a.	n.a.	n.a.	n.a.	25 (30)	39 (46)	36 (43)
Spain	95 (47)	10.1 (4.0)	44 (42)	56 (53)	19.3 (3.8)	4.3 (4)	63.2 (60)	18.9 (18)	13.7 (13)	19 (18)	45 (43)	36 (34)
France	188 (47)	9.6 (3.4)	46 (87)	54 (101)	17.4 (3.6)	14 (26)	71.5 (133)	9.1 (17)	5.4 (10)	n.a.	n.a.	n.a.
Belgium	465 (55)	10.3 (1.2)	32 (150)	68 (315)	18.3 (3.4)	8.4 (38)	66.5 (302)	19.6 (89)	5.5 (25)	n.a.	n.a.	n.a.
UK	201 (56)	10.4 (3.6)	40 (81)	60 (120)	20.1 (6.1)	18.5 (26)	53.2 (75)	14.9 (21)	13.5 (19)	15 (31)	48 (97)	36 (73)
Poland	160 (48)	9.8 (3.4)	46 (74)	54 (86)	17.8 (3.6)	9.1 (1)	72.7 (8)	18.2 (2)	n.a.	8 (12)	59 (94)	34 (54)
Turkey	309 (82)	9.6 (3.6)	48 (148)	52 (161)	18.7 (4.4)	16.5 (51)	53.4 (165)	14.9 (46)	15.2 (47)	n.a.	n.a.	n.a.
Iran	417 (53)	13.0 (3.0)	22 (93)	78 (324)	21.1 (4.6)	6.8 (28)	61.6 (253)	22.6 (93)	9.0 (37)	29 (121)	36 (152)	35 (144)
China	2922 (52)	12.3 (2.7)	20 (580)	80 (2342)	18.3 (3.2)	17.7 (509)	71.1 (2055)	9.2 (265)	2.1 (62)	n.a.	n.a.	n.a.
Indonesia	558 (56)	10.9 (4.1)	37 (206)	63 (352)	n.a.	n.a.	n.a.	n.a.	n.a.	27 (153)	60 (336)	12 (69)
Total population ^b^	6333 (52)	11.4 (3.3)	30 (1930)	70 (4403)	18.9 (3.8)	15.1 (683)	67.8 (3051)	12.2 (551)	4.7 (213)	20 (467)	47 (1086)	32 (739)

Data are presented as percentage (*n*) or mean (95 % CI)

*n.a.* not available, *BMI* body mass index

^a^BMI (kg/m^2^) classification according to IOTF guidelines [8, 9]

^b^Only countries with available data on the presented characteristics were included

^c^Category AB represents individuals with professional/managerial occupations, C represents individuals with other non-manual occupations and individuals having skilled manual occupations, and D represents individuals with semi-/unskilled manual occupations and people dependent on state benefits

China contributed the highest number of males to the total male study sample (Table 1) (*n* = 2705, 47 % of total male study sample) and the lowest Turkey (*n* = 67, 1 % of total male sample). The youngest females were from France (8.9 ± 3.4 years) and the oldest were from Iran (13.0 ± 4.1 years). China provided the highest number of females (Table 2) (*n* = 2922) and Uruguay the lowest (*n* = 71). The youngest females were from Mexico (9.4 ± 3.7 years) and the oldest were from Iran (13.0 ± 3.0 years).

### Total fluid intake

Daily TFI was obtained in 3611 children (4–9 years, 51.75 % females) and 8109 adolescents (10–17 years, 53.11 % females) (Table 3). The highest fluid intake was observed in Uruguayan males (2.13 ± 0.80 and 2.46 ± 1.04 L/day, for 4–9 years and 10–18 years, respectively) and females (2.47 ± 0.92 and 2.61 ± 1.16 L/day, for 4–9 years and 10–18 years, respectively). In contrast, the lowest fluid intake in children and adolescents was observed in Belgian males (0.90 ± 0.43 and 0.99 ± 0.44 L/day, for 4–9 and 10–18 years, respectively) and females (0.73 ± 0.39 and 0.91 ± 0.41 L/day, respectively).

**Table 3:** Daily total fluid intake (the sum of liquids provided by all types of fluids or beverages) (L/day) per age group, sex and country

	Total						Males						Females					
	*n*	Mean	SD	Median	25th	75th	*n*	Mean	SD	Median	25th	75th	*n*	Mean	SD	Median	25th	75th
*Children* (*4–9* *years*)																		
Mexico	387	1.35	0.65	1.23	0.88	1.64	234	1.39	0.65	1.27	0.89	1.71	153	1.28	0.64	1.16	0.84	1.51
Brazil	349	1.67	0.63	1.55	1.20	2.03	183	1.68	0.61	1.55	1.23	2.10	166	1.66	0.66	1.55	1.20	1.94
Uruguay	60	2.24	0.86	2.05	1.70	2.72	30	2.13	0.80	2.00	1.53	2.72	30	2.35	0.92	2.16	1.86	2.78
Argentina	89	1.78	0.92	1.54	1.17	2.03	30	1.64	0.63	1.58	1.23	1.99	59	1.86	1.03	1.50	1.14	2.20
Spain	85	1.64	0.67	1.50	1.13	1.97	43	1.78	0.77	1.68	1.18	2.08	42	1.50	0.53	1.43	1.09	1.73
France	206	1.02	0.35	0.92	0.77	1.22	119	1.02	0.35	0.95	0.77	1.23	87	1.01	0.36	0.88	0.77	1.21
Belgium	266	0.84	0.41	0.81	0.54	1.08	116	0.90	0.43	0.90	0.60	1.16	150	0.79	0.39	0.75	0.49	1.03
UK	148	1.56	0.57	1.51	1.18	1.87	67	1.69	0.64	1.65	1.24	2.08	81	1.45	0.49	1.46	1.13	1.71
Poland	154	1.39	0.44	1.29	1.07	1.64	80	1.42	0.50	1.28	1.06	1.69	74	1.37	0.38	1.29	1.09	1.64
Turkey	164	1.74	0.72	1.67	1.23	2.12	16	1.81	0.75	1.84	1.06	2.31	148	1.73	0.71	1.60	1.23	2.04
Iran	177	1.24	0.42	1.19	0.95	1.46	84	1.32	0.44	1.25	1.02	1.47	93	1.16	0.39	1.13	0.87	1.41
China	1120	0.97	0.43	0.89	0.64	1.19	540	0.98	0.42	0.90	0.65	1.20	580	0.95	0.43	0.86	0.63	1.17
Indonesia	406	1.88	0.77	1.73	1.26	2.44	200	1.94	0.78	1.82	1.36	2.55	206	1.83	0.77	1.68	1.19	2.29
TOTAL	3611	1.32	0.68	1.18	0.84	1.64	1742	1.34	0.67	1.20	0.86	1.67	1869	1.30	0.69	1.16	0.81	1.62
*Adolescents* (*10–18* *years*)																		
Mexico	306	1.51	0.85	1.31	0.91	1.91	172	1.46	0.74	1.33	0.96	1.81	134	1.57	0.97	1.29	0.84	2.05
Brazil	430	2.01	0.90	1.80	1.40	2.54	212	2.03	0.92	1.89	1.35	2.55	218	1.99	0.88	1.75	1.40	2.48
Uruguay	79	2.54	1.04	2.23	1.75	3.16	38	2.46	0.92	2.21	1.77	3.04	41	2.61	1.16	2.25	1.75	3.42
Argentina	104	1.77	0.81	1.59	1.21	2.11	44	1.83	0.97	1.57	1.17	2.24	60	1.72	0.68	1.63	1.26	2.07
Spain	116	1.78	0.70	1.62	1.33	2.14	63	1.80	0.64	1.63	1.36	2.15	53	1.75	0.76	1.57	1.27	2.15
France	193	1.25	0.45	1.16	0.93	1.47	92	1.35	0.46	1.28	1.04	1.57	101	1.17	0.43	1.07	0.91	1.32
Belgium	574	0.95	0.43	0.91	0.69	1.14	259	0.99	0.44	0.95	0.71	1.18	315	0.91	0.41	0.86	0.65	1.09
UK	210	1.67	0.73	1.55	1.15	2.09	90	1.77	0.75	1.61	1.23	2.16	120	1.60	0.72	1.45	1.10	1.96
Poland	176	1.48	0.48	1.41	1.12	1.73	90	1.44	0.46	1.42	1.10	1.69	86	1.51	0.51	1.39	1.14	1.93
Turkey	212	1.91	0.80	1.77	1.26	2.46	51	1.98	0.86	1.91	1.16	2.77	161	1.88	0.78	1.72	1.31	2.34
Iran	607	1.32	0.51	1.26	0.94	1.58	283	1.42	0.53	1.36	1.00	1.70	324	1.23	0.47	1.16	0.86	1.52
China	4507	1.15	0.54	1.05	0.76	1.43	2165	1.24	0.57	1.13	0.81	1.53	2342	1.08	0.49	0.99	0.72	1.33
Indonesia	595	2.03	0.83	1.91	1.37	2.66	243	2.05	0.86	1.97	1.34	2.75	352	2.01	0.81	1.90	1.38	2.62
Total	8109	1.35	0.71	1.19	0.84	1.65	3802	1.40	0.71	1.25	0.90	1.73	4307	1.30	0.70	1.13	0.80	1.59

### Adherence to EFSA reference values

**Fig. 1:**
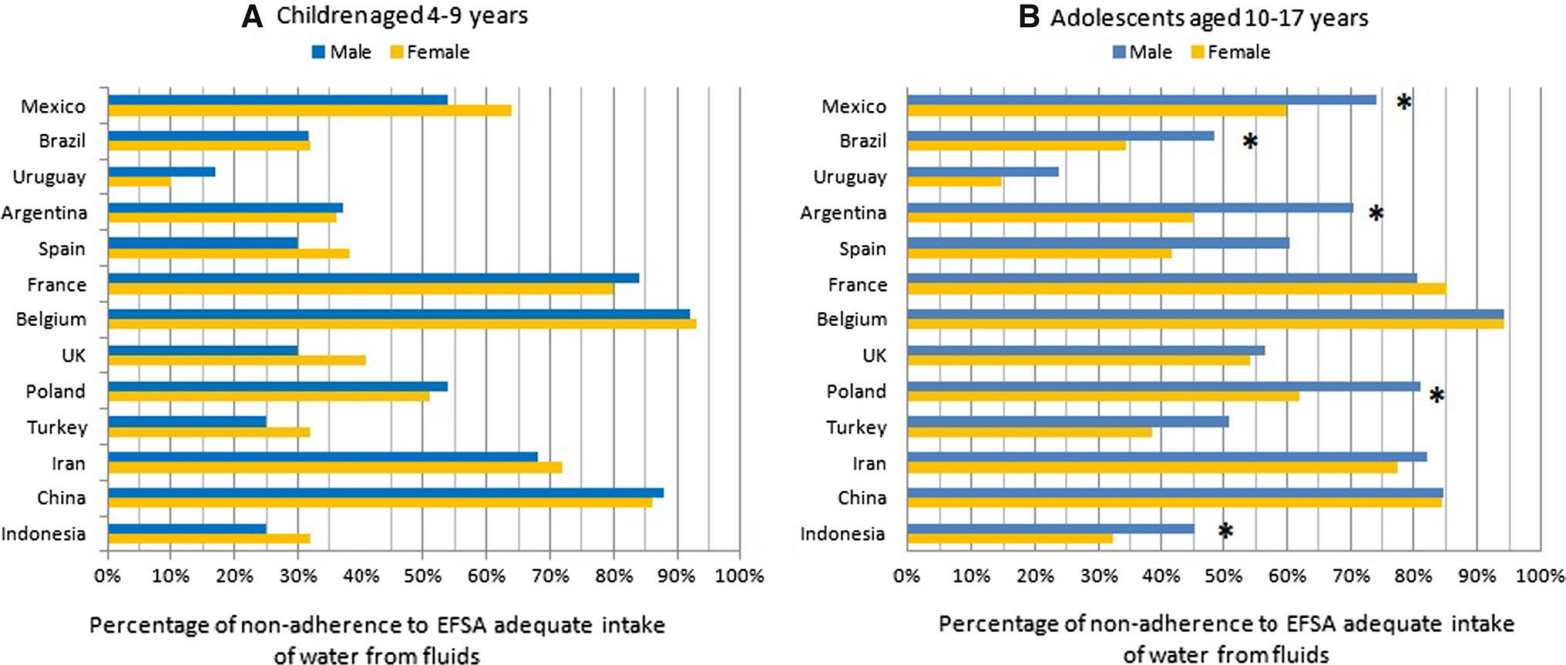
Non-adherence to EFSA adequate intake for fluids (equals 80 % of EFSA adequate intake for total water intake) by age group. *p value of the Pearson Chi-square test < 0.05 (differences between sexes within countries)

The non-adherence to EFSA AIs for fluids in children and adolescents is shown in Fig. 1. A high proportion of the participants do not meet the EFSA AIs for fluids. In children, the lowest non-adherence was observed in Uruguay, males (10 %) and females (<20 %), matching with the country which provided fewer participants, and the highest non-adherence was observed in Belgium, males and females (>90 %). No significant differences for adherence were observed between males and females in all countries.

In adolescents, the lowest non-adherence to EFSA AIs for fluids was observed in Uruguay (males and females <25 and 15 %, respectively) and the highest non-adherence was observed in Belgium (males and females >90 %). Non-adherence to EFSA AIs for fluids was significantly higher in males than in females in Indonesia, Brazil, Argentina, Mexico and Poland.

**Fig. 2:**
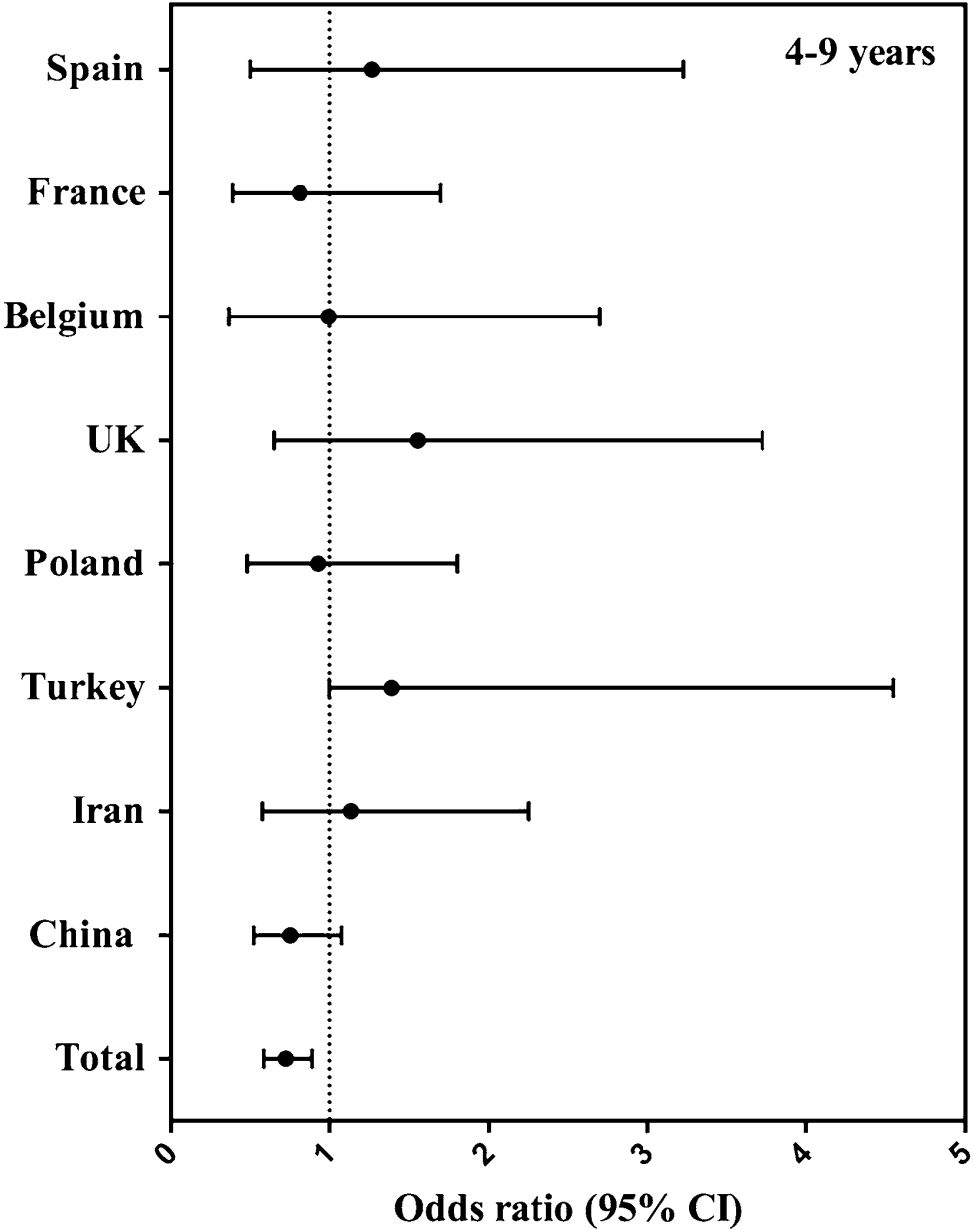
Association (odds ratio, 95 % CI) between compliance with EFSA adequate intake for fluids (equals 80 % of EFSA adequate intake for total water intake) and gender, per country in children aged 4–9 years. The logistic regression model was adjusted for age, BMI and SEL, and males were considered as reference. *p value < 0.05

**Fig. 3:**
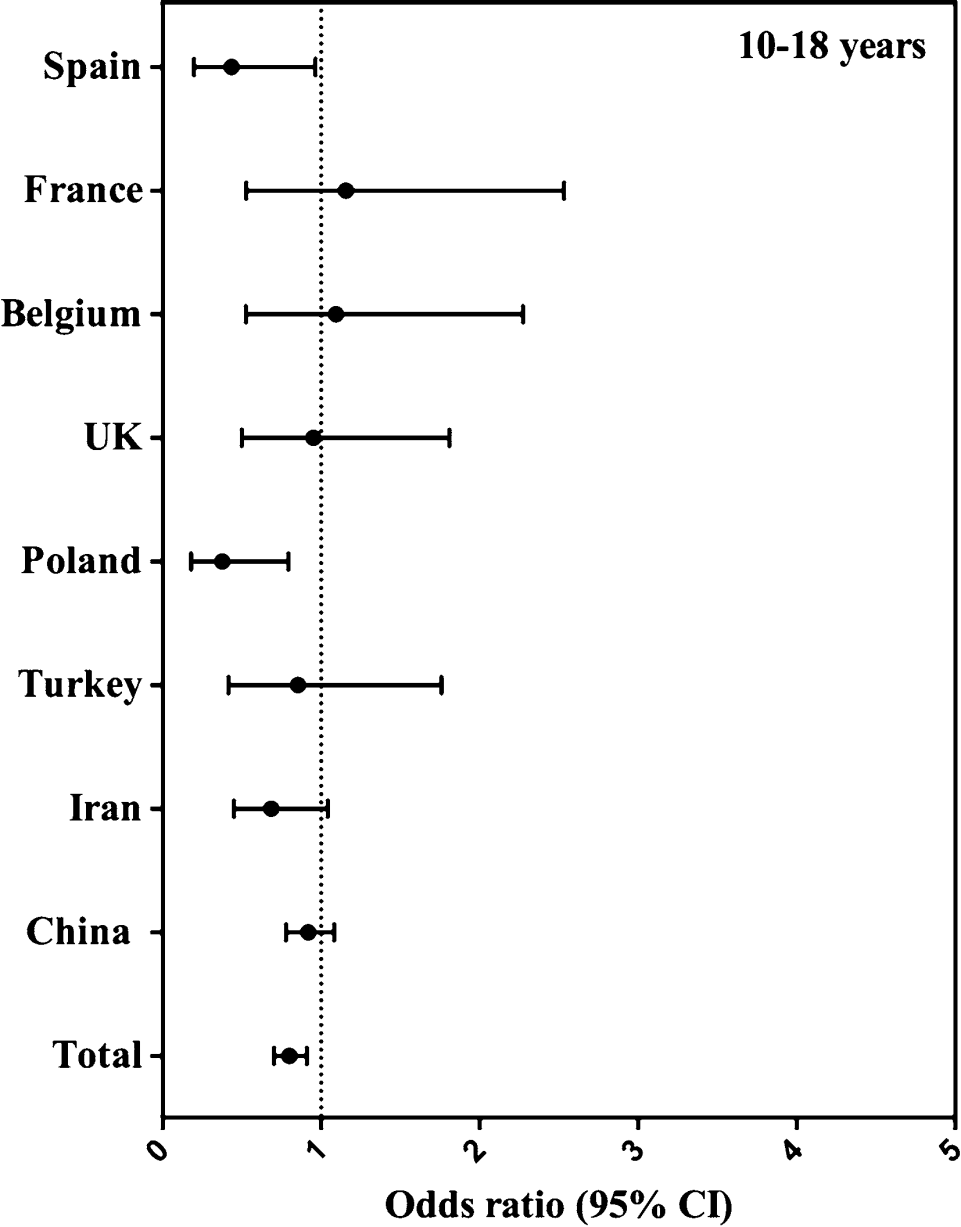
Association (odds ratio, 95 % CI) between compliance with EFSA adequate intake for fluids (equals 80 % of EFSA adequate intake for total water intake) and gender, per country in adolescents aged 10–17 years. The logistic regression model was adjusted for age, BMI and SEL, and males were considered as reference

In Figs. 2 and 3, compliance with EFSA AIs for fluids (yes/no) is considered as the dependent variable and gender as the independent variable. The odds ratios (OR) represented the likelihood of not reaching the EFSA AIs for fluids when being female as compared to being male. In the total sample, females were more likely to meet the EFSA AIs for fluids than the males (4–9 years: 28 %, OR 0.72, *p* = 0.002; 10–18 years: 20 %, OR 0.80, *p* = 0.001). When analysing the samples of the countries individually, the probability of not meeting the EFSA AIs for fluids reached significance only in Spain and Poland, and moreover only in the age group 10–18 years: females were more likely to meet the EFSA AIs for fluids than males (Spain: 57 %, OR 0.43, *p* = 0.041; and Poland: 62 %, OR 0.38, *p* = 0.010).

**Fig. 4:**
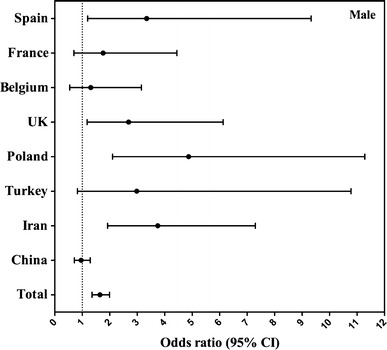
Association (odds ratio, 95 % CI) between compliance with EFSA adequate intake for fluids (equals 80 % of EFSA adequate intake for total water intake) and age group per country in males. The logistic regression model was adjusted for BMI and SEL, and children aged 4–9 were considered as reference

**Fig. 5:**
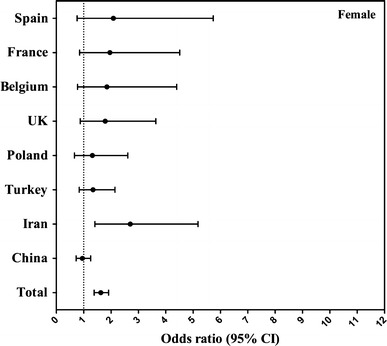
Association (odds ratio, 95 % CI) between compliance with EFSA adequate intake for fluids (equals 80 % of EFSA adequate intake for total water intake) and age group per country in females. The logistic regression model was adjusted for BMI and SEL, and children aged 4–9 were considered as reference

In Figs. 4 and 5, compliance with EFSA recommendation for TFI (yes/no) is considered as dependent variable and age 4–9 as independent variable. The odds ratio (OR) represents the percentage not reaching the EFSA recommendation for TFI when being older than 9 years.

Differences were found between country and gender (males Fig. 4; females Fig. 5). The probability of non-compliance with EFSA AI for fluids increased significantly when being older than 9 years in Spain (OR 3.345, *p* = 0.021 in males), UK (OR 2.689, *p* = 0.019, in males), Poland (OR 4.871, *p* < 0.001 in males), Iran (OR 3.747, *p* < 0.001 in males and OR 2.702, *p* = 0.003 in females) and the total sample (OR 1.645, *p* < 0.001 in males and OR 1.625, *p* < 0.001 in females). In the rest of the countries except for China, children older than 9 years were more likely to comply with the EFSA AIs; however, this was not significant. In China, the probability of not compliance with EFSA recommendation was lower when being older than 9 years in both genders, although this did not reach significance.

## Discussion

The results of this compilation of cross-sectional studies provide important information about the fluid intake of children in 13 countries, and such a global perspective has not previously been reported. According to the EFSA reference values for total water intake for these age categories, more than fifty per cent of the whole study population are at risk of an inadequate intake.

Large differences between countries and age groups were found. Belgium had the highest percentage (>90 %) of non-adherence to the EFSA AIs for fluids in both children and adolescents, followed by China, France (both >80 %) and Iran (70 %). In Belgium, China and Iran, the completion of the fluid diary was done by the teachers in the school and by the parents at home, whereas in the rest of the countries, the diary was filled by main caretaker (mums). This fact may explain the lower intakes recorded in the firsts countries. Maybe the intake in these other countries was overestimated because of social desirability and is the intake of Belgium actually more accurately reflecting the true intake. In France, a study [6] with a representative sample of children and adolescents suggested that fluid intake was low (1–1.1 L/day), very similar to the values obtained in the present study. Moreover, another study demonstrated that based on osmolality urine levels, two-thirds of French children had a hydration deficit in the morning when they went to school, despite having breakfast [7]. Both these studies support our findings of an inadequate fluid intake in French children. In Europe, data from the HELENA study [15] performed in adolescents, and the German DONALD study [26] performed in both children and adolescents, suggested higher values of fluid intakes than our study did. However, the tool used in the HELENA study was not specifically developed to measure fluid intake [15]. In addition, the software used in the HELENA study to collect the 24-h recalls (HELENA-DIAT [11]) was designed to remind the adolescents to fulfil the questionnaire to state the amount of water intake as is an issue susceptible to oversight such as other add-ons like butter, or salad dressing.

Uruguay (<20 %) and Brazil (<40 %) had the lowest percentages of non-adherents to EFSA AIs for fluids. Together with Argentina, Turkey and Indonesia, Uruguay had the highest mean fluid intakes, similar to the values observed in a previous study in Brazil [18]. One of the plausible explanations for this finding could be the high humidity and temperatures in these countries, which result in people drinking more [30]. This raises one of the limitations of present analysis of cross-sectional surveys. The fact that the surveys were performed in the same period of the year, and therefore the impact of the seasons on TFI was neglected. Nevertheless, the data collection was performed during the spring or autumn, periods expected to have a mild climate. The impact of climate on TFI should be investigated by recording the temperature and humidity in future surveys and considered in analyses.

One remarkable point throughout this analysis is that there were no large differences in fluid consumption between the genders. Generally males drank more fluids than females in all the countries included in this survey (except females from Uruguay and Argentina aged 4–10 years, and females from Mexico and Uruguay aged 10–18 years). However, females were more likely to comply with EFSA AIs for fluids. Gender differences were more noticeable in the adolescents throughout the countries and in the total sample, probably because at these ages, females become more health conscious and may drink more [23].

The results also indicated that compliance with EFSA AIs for fluids is greater among children 4–9 years old than among children 10–18 years old in almost all included countries. A plausible explanation could be that with transition to adolescence children gain more independence with respect to food/fluid consumption. While they still consume the majority of meals and snacks at home, but they also start taking decisions regarding food choices away from home [22]. With the transition to adolescence, a possible transition in the preference for certain beverage types might be anticipated. This topic was, however, outside the scope of the current paper, but will be addressed in a separate paper [20].

Several limitations should be considered when interpreting the data presented. Firstly, the lack of representativeness of the samples in terms of “*n*”, age groups and gender within the countries suggests that these data should be interpreted cautiously: as country-, age- and gender-specific data points. Secondly, the lacks of data regarding BMI or SEL in some of the countries did not allow having a complete set up of the situation. Another limitation to consider is related to the method used for recording fluid intake: for children younger than 12 years, the primary caretaker was responsible to record the fluid intake of the child. It should be considered that a parent might find it difficult to accurately estimate fluid intake consumed at school. Therefore, fluid intake of the children/adolescent could be over- or underreported. However, fluid intake of all participants was recorded over a period of 7 days including the weekend during which the children are at home and the caregiver can observe and record the intake of their child more carefully. Future research should focus on demonstrating the health impact of a certain level of fluid intake, in order to progress towards a reference value based on scientific evidence and not only on observed intakes. Regardless of which cut-off was used to evaluate the wide of the problem (meet/not meet water intake recommendations), the observation from these cross-sectional surveys remains the same: that a high number of children and adolescents worldwide have a low TFI. For example, when using the dietary reference values set in China (which use those established by the Institute of Medicine—IOM—in USA [2]), 86 % of both males and females were at risk of an inadequate water intake, which is a very similar percentage to the ones we have obtained based on the EFSA recommendations even when the values established by the IOM are higher than the ones from EFSA. This is because there are almost no subjects located between the EFSA reference values and IOM ones, regarding drinking fluids.

The strengths of all 13 cross-sectional surveys include the use of a standardized 7-day fluid-specific record, considered the reference method to assess fluid intake and the amount of water volumes also in young population groups [31, 32]. This fluid-specific record was also supported by visual aids, to facilitate the recording of consumed volumes. Moreover, the two methods used for the recruitment (the quota-based sample—the best method to evaluate intakes [19]—and stratified clusterization) used to approach the survey in 10 and in the other three countries, respectively, are recognized as valuable methods to provide enough sample by age of participants, regions of the country and different socio-economic groups for meaningful analysis.

## Conclusions

The most important conclusions from this analysis are that a high proportion of children and adolescents are at risk of an inadequate fluid intake and that the probability for non-adherence to EFSA AIs for fluids is higher among males than among females, and among those aged from 4 to 9 than among those aged from 10 to 18. Uruguay followed by Brazil and Belgium followed by China were those countries with the highest proportion of adherents and non-adherents, respectively, to EFSA AIs for fluids. As a number of studies have demonstrated that having a low fluid or water intake can compromise several body functions, this conclusion justifies encouraging these populations to increase their plain water intake. Future studies should focus on the observation of longitudinal changes to determine whether the maintained restrictive water intake can result in long-term health impacts from the early stages of life.

## Electronic supplementary material

ONLINE RESOURCE 1: Survey description (DOCX 25 kb)

ONLINE RESOURCE 2: Percentile of total fluid intake of children and adolescents (DOCX 23 kb)
