# Supplementary material for: Total fluid intake of children and adolescents: cross-sectional surveys in 13 countries worldwide
Source: Eur J Nutr. 2015 Jun 18;54(Suppl 2):57–67. doi: 10.1007/s00394-015-0946-6 (PMC4473088; doi:10.1007/s00394-015-0946-6)
Supplement: Supplementary file 1 — ONLINE RESOURCE 1: Survey description (DOCX 25 kb) [file 394_2015_946_MOESM1_ESM.docx]

**European Journal of Nutrition**

**Total fluid intake of children and adolescents: cross sectional surveys in 13 countries worldwide**

*Iglesia I*^1,2^*, Guelinckx I^3^, De Miguel-Etayo P* ^1,2^*, González-Gil E.M.* ^1,2^*, Salas-Salvadó J^4,5^*, *Kavouras SA^6^, Gandy J^7^, Martinez H^8,9^ , Bardosono S^9,10^, Abdollahi M^11^, Nasseri E^12^ , Jarosz A^12^, Ma G^13,14^, Carmuega E^15^, Thiébaut I^16,1^, Moreno LA^1,2^*

**Corresponding author**

Prof. Luis A. Moreno, University of Zaragoza, Faculty of Health Sciences

C/ Domingo Miral s/n, 50009 Zaragoza, Spain

Tel: +34 876554457; Fax: +34 976761720; e-mail: [lmoreno@unizar.es](mailto:lmoreno@unizar.es)

Online Resource 1. General characteristics (a) and data collection (b) of 1713 cross sectional surveys aiming to specifically record intake of fluids (water and beverages of all kind)

| 1. General characteristics of cross-sectional surveys | | | | | | | | | |
| --- | --- | --- | --- | --- | --- | --- | --- | --- | --- |
| Country, study year | **Responsible Institute** | **Sampling method** | **If a quota based method or stratification was used, quotas/stratification were set for:** | | | | | | **Exclusion criteria** |
|  |  |  | Age | Sex | Regions | Urban/ rural area | SEL | Other |  |
| Mexico, 2012 | IPSOS | Systematic random sampling, Quota method | Yes | Yes | Yes | No | Yes | No | None |
| Brazil, 2008 | GFK | Quota method | Yes | Yes | Yes | No | Yes | No | Working in company advertising ^a^ |
| Argentina, 2009 | TNS | Quota method | Yes | Yes | Yes | No | Yes | No | Working in company advertising ^a^; participating to survey about non-alcoholic drinks in last 6 months |
| Uruguay, 2012 | GFK | Quota method | Yes | Yes | Yes | No | Yes | No | Working in company advertising ^a^ |
| Spain, 2012 | TNS | Quota method | Yes | Yes | Yes | Yes | Yes | Educational level | Working in company advertising ^a^ |
| France, 2012 | TNS | Quota method | Yes | Yes | Yes | No | Yes | No | Working in company advertising ^a^ |
| Belgium, 2012 | CEDE | Random, stratified Cluster sampling (schools) | Yes | Yes | Yes | No | No | Educational system | None |
| UK, 2010 | IPSOS | Quota method | Yes | Yes | Yes | No | Yes | No | Away from home for more than two nights in the sampling week ; specific diagnosed disease; following a medically-prescribed diet |
| Germany, 2012 | TNS | Quota method | Yes | Yes | Yes | No | Yes | Educational level | Working in company advertising ^a^ |
| Poland, 2014 | TNS | Quota method | Yes | Yes | Yes | Yes | Yes | Educational level | Working in company advertising ^a^; participating in market research within last 6 months |
| Turkey, 2011 | IPSOS | Systematic random sampling, Quota method | Yes | Yes | Yes | No | Yes | No | None |
| Iran, 2011 | NNFTRI | Quota method | Yes | Yes | Yes | No | Yes | No | specific diagnosed disease; following a medically-prescribed diet |
| Iran, 2013 | NNFTRI | Random, stratified Cluster sampling (schools) | No | Yes | Yes | No | Yes | School grade | None |
| China, 2010 | CDC | Multi-stage random sampling | Yes | Yes | Yes | No | No | No | specific diagnosed disease |
| China, 2011 | CDC | Multi-stage random sampling | No | No | No | Yes | No | School grade | specific diagnosed disease |
| Indonesia, 2012 | Nielsen | Systematic random sampling, Quota method | Yes | Yes | No | Yes | Yes | No | Working in company advertising ^a^ |
| Japan, 2009 | IPSOS | Quota method | Yes | Yes | Yes | No | No | No | Working in company advertising ^a^; participating in market research within last 3 months |

^a^ Working in company advertising, marketing, market research, media or manufacture, distribution and sale of different types of beverages

Abbreviations: CEDE Club Européen des Diététiciens de l’Enfance; n.a. not applicable; NNFTRI National Nutrition and FoodTechnology Research Institute, Shahid Beheshti University of Medical Sciences, Tehran, Iran; SEL socio economical level.

| 1. Data collection | | | | | | |
| --- | --- | --- | --- | --- | --- | --- |
| Country | **Period of data collection** | **Age range** | **Age recording** | **Dietary assessment method** | **Administration form** | **Antropometric data** |
| Mexico, 2012 | April – May | 1-65 | Continuous | 7 day fluid record | Paper | n.a. |
| Brazil, 2008 | Wave 1: March Wave 2: September | 1-55 | Continuous | 2 day fluid record | Paper | n.a. |
| Argentina, 2012 | November - December | 0-65 | Continuous | 7 day fluid record ^b^ | Paper | n.a. |
| Uruguay, 2012 | Wave 1: March Wave 2: December | 6-65 | Categorical | 7 day fluid record | Paper | n.a. |
| Spain, 2012 | March - May | 6-70 | Continuous | 7 day fluid record | Paper | Self-reported |
| France, 2012 | April | 0-70 | Continuous | 7 day fluid record | Online | Self-reported |
| Belgium, 2012 | February - June | 8-13 | Continuous | 7 day fluid record | Paper | Measured |
| UK, 2010 | May | 3-65 | Continuous | 7 day fluid record | Paper | Self-reported |
| Germany, 2012 | April | 16-65 | Continuous | 7 day fluid record | Online | Self-reported |
| Poland, 2014 | May | 3-87 | Continuous | 7 day fluid record | Paper | Measured |
| Turkey, 2011 | January – February | 2-55 | Continuous | 7 day fluid record | Paper | Self-reported |
| Iran, 2011 | May – June | 18 - 65 | Continuous | 7 day fluid record ^b^ | Paper | Measured |
| Iran, 2013 | April – May | 8 - 17 | Continuous | 7 day fluid record ^b^ | Paper | Measured |
| China, 2010 | June – July | 18 - 60 | Continuous | 7 day fluid record | Paper | Measured |
| China, 2011 | September – October | 8 – 17 | Continuous | 7 day fluid record | Paper | Measured |
| Indonesia, 2012 | June – July | 1- 65 | Continuous | 7 day fluid record | Paper | Self-reported |
| Japan, 2009 | June | 18 - 60 | Categorical | 7 day fluid record | Online | n.a. |

^b^ Non-alcoholic beverages only
