# Supplementary material for: Total fluid intake of children and adolescents: cross-sectional surveys in 13 countries worldwide
Source: Eur J Nutr. 2015 Jun 18;54(Suppl 2):57–67. doi: 10.1007/s00394-015-0946-6 (PMC4473088; doi:10.1007/s00394-015-0946-6)
Supplement: Supplementary file 2 — ONLINE RESOURCE 2: Percentile of total fluid intake of children and adolescents (DOCX 23 kb) [file 394_2015_946_MOESM2_ESM.docx]

**European Journal of Nutrition**

**Total fluid intake of children and adolescents: cross sectional surveys in 13 countries worldwide**

*Iglesia I*^1,2^*, Guelinckx I^3^, De Miguel-Etayo P* ^1,2^*, González-Gil E.M.* ^1,2^*, Salas-Salvadó J^4,5^*, *Kavouras SA^6^, Gandy J^7^, Martinez H^8,9^ , Bardosono S^9,10^, Abdollahi M^11^, Nasseri E^12^ , Jarosz A^12^, Ma G^13,14^, Carmuega E^15^, Thiébaut I^16,1^, Moreno LA^1,2^*

**Corresponding author**

Prof. Luis A. Moreno, University of Zaragoza, Faculty of Health Sciences

C/ Domingo Miral s/n, 50009 Zaragoza, Spain

Tel: +34 876554457; Fax: +34 976761720; e-mail: [lmoreno@unizar.es](mailto:lmoreno@unizar.es)

^b^ Non-alcoholic beverages only

Online Resource 2. Percentiles of daily total fluid intake (L/day) of children (4-9 years) and adolescents (10-17 years) according to age and sex

|  | *Total sample* | | | | | *Boys* | | | | | *Girls* | | | | |
| --- | --- | --- | --- | --- | --- | --- | --- | --- | --- | --- | --- | --- | --- | --- | --- |
|  |  | *Percentiles* | | | |  | *Percentiles* | | | |  | *Percentiles* | | | |
|  | *SEM* | *5* | *10* | *90* | *95* | *SEM* | *5* | *10* | *90* | *95* | *SEM* | *5* | *10* | *90* | *95* |
| Children |  |  |  |  |  |  |  |  |  |  |  |  |  |  |  |
| Mexico | 0,03 | 0,59 | 0,67 | 2,09 | 2,55 | 0,04 | 0,61 | 0,67 | 2,18 | 2,57 | 0,05 | 0,57 | 0,64 | 2,04 | 2,54 |
| Brazil | 0,03 | 0,80 | 0,98 | 2,50 | 2,97 | 0,05 | 0,90 | 1,00 | 2,51 | 2,92 | 0,05 | 0,74 | 0,92 | 2,51 | 3,15 |
| Uruguay | 0,11 | 1,05 | 1,28 | 3,35 | 3,58 | 0,15 | 1,05 | 1,14 | 3,31 | 3,70 | 0,17 | 1,03 | 1,43 | 3,57 | 4,47 |
| Argentina | 0,10 | 0,83 | 0,98 | 3,08 | 3,55 | 0,12 | 0,66 | 0,97 | 2,46 | 3,22 | 0,13 | 0,82 | 1,00 | 3,30 | 4,44 |
| Spain | 0,07 | 0,82 | 0,98 | 2,56 | 3,08 | 0,12 | 0,81 | 0,96 | 2,84 | 3,52 | 0,08 | 0,80 | 0,98 | 2,11 | 2,78 |
| France | 0,02 | 0,59 | 0,64 | 1,53 | 1,71 | 0,03 | 0,59 | 0,63 | 1,58 | 1,71 | 0,04 | 0,60 | 0,67 | 1,52 | 1,75 |
| Belgium | 0,03 | 0,21 | 0,34 | 1,40 | 1,58 | 0,04 | 0,21 | 0,36 | 1,46 | 1,64 | 0,03 | 0,21 | 0,32 | 1,34 | 1,51 |
| UK | 0,05 | 0,78 | 0,82 | 2,27 | 2,57 | 0,08 | 0,79 | 0,92 | 2,55 | 3,00 | 0,05 | 0,76 | 0,81 | 2,16 | 2,25 |
| Poland | 0,04 | 0,83 | 0,90 | 2,02 | 2,25 | 0,06 | 0,82 | 0,90 | 2,17 | 2,49 | 0,04 | 0,84 | 0,90 | 1,82 | 2,04 |
| Turkey | 0,06 | 0,75 | 0,95 | 2,78 | 3,30 | 0,19 | 0,71 | 0,72 | 2,91 | 3,27 | 0,06 | 0,84 | 0,98 | 2,80 | 3,31 |
| Iran | 0,03 | 0,64 | 0,75 | 1,82 | 1,99 | 0,05 | 0,68 | 0,82 | 1,95 | 2,25 | 0,04 | 0,58 | 0,69 | 1,67 | 1,88 |
| China | 0,01 | 0,45 | 0,50 | 1,50 | 1,79 | 0,02 | 0,46 | 0,52 | 1,53 | 1,81 | 0,02 | 0,44 | 0,49 | 1,50 | 1,77 |
| Indonesia | 0,04 | 0,82 | 0,96 | 3,03 | 3,33 | 0,05 | 0,80 | 0,97 | 3,06 | 3,29 | 0,05 | 0,83 | 0,95 | 3,00 | 3,36 |
| TOTAL | 0,01 | 0,49 | 0,60 | 2,21 | 2,67 | 0,02 | 0,52 | 0,62 | 2,26 | 2,67 | 0,02 | 0,48 | 0,58 | 2,15 | 2,67 |
| Adolescents |  |  |  |  |  |  |  |  |  |  |  |  |  |  |  |
| Mexico | 0,05 | 0,57 | 0,67 | 2,53 | 3,36 | 0,06 | 0,57 | 0,67 | 2,46 | 2,98 | 0,08 | 0,60 | 0,67 | 2,63 | 3,80 |
| Brazil | 0,04 | 0,88 | 1,01 | 3,32 | 3,74 | 0,06 | 0,87 | 0,98 | 3,44 | 3,80 | 0,06 | 0,85 | 1,05 | 3,28 | 3,66 |
| Uruguay | 0,12 | 1,10 | 1,49 | 4,09 | 4,58 | 0,15 | 1,31 | 1,50 | 3,62 | 4,25 | 0,18 | 0,88 | 1,16 | 4,36 | 5,09 |
| Argentina | 0,08 | 0,90 | 0,99 | 2,84 | 3,61 | 0,15 | 0,91 | 0,98 | 3,33 | 3,92 | 0,09 | 0,85 | 0,99 | 2,60 | 3,33 |
| Spain | 0,06 | 0,83 | 1,08 | 2,67 | 3,24 | 0,08 | 1,01 | 1,18 | 2,74 | 3,20 | 0,10 | 0,77 | 0,93 | 2,64 | 3,39 |
| France | 0,03 | 0,66 | 0,77 | 1,85 | 2,25 | 0,05 | 0,69 | 0,82 | 1,97 | 2,33 | 0,04 | 0,64 | 0,77 | 1,71 | 2,20 |
| Belgium | 0,02 | 0,36 | 0,47 | 1,44 | 1,69 | 0,03 | 0,35 | 0,47 | 1,56 | 1,73 | 0,02 | 0,36 | 0,46 | 1,36 | 1,58 |
| UK | 0,05 | 0,74 | 0,88 | 2,73 | 3,20 | 0,08 | 0,78 | 0,95 | 2,91 | 3,44 | 0,07 | 0,73 | 0,83 | 2,52 | 3,06 |
| Poland | 0,04 | 0,79 | 0,89 | 2,27 | 2,42 | 0,05 | 0,76 | 0,83 | 2,21 | 2,36 | 0,05 | 0,82 | 0,92 | 2,33 | 2,49 |
| Turkey | 0,06 | 0,80 | 0,91 | 3,08 | 3,39 | 0,12 | 0,73 | 0,90 | 3,16 | 3,45 | 0,06 | 0,80 | 0,91 | 3,08 | 3,35 |
| Iran | 0,02 | 0,66 | 0,73 | 2,01 | 2,24 | 0,03 | 0,73 | 0,85 | 2,13 | 2,34 | 0,03 | 0,60 | 0,68 | 1,89 | 2,09 |
| China | 0,01 | 0,50 | 0,57 | 1,87 | 2,21 | 0,01 | 0,53 | 0,60 | 2,03 | 2,35 | 0,01 | 0,49 | 0,55 | 1,69 | 1,97 |
| Indonesia | 0,03 | 0,84 | 0,98 | 3,26 | 3,49 | 0,05 | 0,84 | 1,00 | 3,27 | 3,60 | 0,04 | 0,84 | 0,95 | 3,26 | 3,45 |
| TOTAL | 0,01 | 0,53 | 0,62 | 2,29 | 2,79 | 0,01 | 0,55 | 0,66 | 2,34 | 2,83 | 0,01 | 0,51 | 0,60 | 2,24 | 2,76 |
